# Automatic Reproduction of Natural Head Position in Orthognathic Surgery Using a Geometric Deep Learning Network

**DOI:** 10.3390/diagnostics15010042

**Published:** 2024-12-27

**Authors:** Ji-Yong Yoo, Su Yang, Sang-Heon Lim, Ji Yong Han, Jun-Min Kim, Jo-Eun Kim, Kyung-Hoe Huh, Sam-Sun Lee, Min-Suk Heo, Hoon Joo Yang, Won-Jin Yi

**Affiliations:** 1 Department of Biomedical Radiation Sciences, Graduate School of Convergence Science and Technology, Seoul National University, Seoul 08826, Republic of Korea; gd_yoo@snu.ac.kr; 2 Department of Applied Bioengineering, Graduate School of Convergence Science and Technology, Seoul National University, Seoul 08826, Republic of Korea; s8431@snu.ac.kr; 3 Interdisciplinary Program in Bioengineering, Graduate School of Engineering, Seoul National University, Seoul 08826, Republic of Korea; smion123@snu.ac.kr (S.-H.L.); jiyong@snu.ac.kr (J.Y.H.); 4 Department of Electronics and Information Engineering, Hansung University, Seoul 02876, Republic of Korea; jmkim@hansung.ac.kr; 5 Department of Oral and Maxillofacial Radiology, Dental Research Institute, School of Dentistry, Seoul National University, Seoul 03080, Republic of Korea; noel1st@snu.ac.kr (J.-E.K.); future3@snu.ac.kr (K.-H.H.); raylee@snu.ac.kr (S.-S.L.); hmslsh@snu.ac.kr (M.-S.H.); 6 Department of Oral and Maxillofacial Surgery, Dental Research Institute, School of Dentistry, Seoul National University, Seoul 03080, Republic of Korea

**Keywords:** orthognathic surgery, computed tomography, natural head position, head pose estimation, geometric deep learning

## Abstract

**Background**: Accurate determination of the natural head position (NHP) is essential in orthognathic surgery for optimal surgical planning and improved patient outcomes. However, traditional methods encounter reproducibility issues and rely on external devices or patient cooperation, potentially leading to inaccuracies in the surgical plan. **Methods**: To address these limitations, we developed a geometric deep learning network (NHP-Net) to automatically reproduce NHP from CT scans. A dataset of 150 orthognathic surgery patients was utilized. Three-dimensional skull meshes were converted into point clouds and normalized to fit within a unit sphere. NHP-Net was trained to predict a 3 × 3 rotation matrix to align the CT-acquired posture with the NHP. Experiments were conducted to determine optimal point cloud sizes and loss functions. Performance was evaluated using mean absolute error (MAE) for roll, pitch, and yaw angles, as well as a rotation error (RE) metric. **Results**: NHP-Net achieved the lowest RE of 1.918° ± 1.099° and demonstrated significantly lower MAEs in roll and pitch angles compared to other deep learning models (*p* < 0.05). These findings indicate that NHP-Net can accurately align CT-acquired postures to the NHP, enhancing the precision of surgical planning. **Conclusions**: By effectively improving the accuracy and efficiency of NHP reproduction, NHP-Net reduces the workload of surgeons, supports more precise orthognathic surgical interventions, and ultimately contributes to better patient outcomes.

## 1. Introduction

Orthognathic surgery necessitates the accurate determination of the natural head position (NHP) as it is a fundamental reference posture extensively utilized in this field [1]. NHP represents the patient’s most natural and comfortable head position, providing a consistent baseline for assessing and diagnosing facial and cranial asymmetries [2,3,4,5,6]. This consistent baseline is essential for optimizing surgical outcomes by offering a standardized reference point, aiding in precise surgical planning, predicting post-operative results, and enhancing communication among dental professionals [7]. Consequently, the accurate determination of NHP is critical for achieving successful surgical interventions and improving patient outcomes in craniofacial analysis [6].

**Figure 1:**
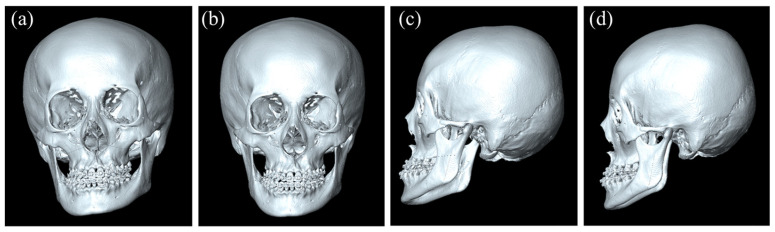
Three-dimensional skull mesh from CT scans before NHP reproduction (**a**) and after NHP reproduction (**b**) in the front view. Three-dimensional skull mesh from CT scans before NHP reproduction (**c**) and after NHP reproduction (**d**) in the side view.

Misalignment in NHP can lead to suboptimal surgical outcomes and patient dissatisfaction. Achieving the correct head position involves defining NHP as the upright head position with eyes fixating on a distant point at eye level, serving as the ideal reference posture [5]. Figure 1 shows 3D skull meshes from CT scans before and after NHP reproductions for the patient with facial asymmetry, highlighting the importance of accurate NHP determination in surgical planning. NHP is particularly important for patients with facial asymmetries, as it ensures the surgical corrections are aligned with the patient’s natural anatomical orientation. The importance of NHP extends beyond surgical procedures; it also plays a vital role in pre-operative planning and post-operative assessment, where even slight deviations can significantly impact the results and patient satisfaction [8].

However, the current methods for NHP reproduction have several limitations and inconveniences that impact their effectiveness and practicality. First, there is a lack of reproducibility due to individual variations and the difficulty for patients to maintain a consistent natural posture [9]. Patients may unintentionally alter their head position between measurements, leading to inconsistencies that can affect diagnostic accuracy and treatment outcomes. Second, traditional intracranial landmarks, such as the sella-nasion line and Frankfort horizontal plane [3,10,11], can be unreliable, especially in patients with significant craniofacial deformities [12,13,14], as these landmarks may not accurately reflect true anatomical orientation. Such discrepancies may lead to misalignment of surgical templates and inaccurate cephalometric measurements, which are essential for precise surgical planning. Deviations in NHP as small as 1–2° can result in cephalometric errors of 2–3 mm, leading to intraoperative adjustments, prolonged surgical time, and increased patient burden. This not only reduces surgical precision but also negatively impacts patient satisfaction [5,11,15,16,17]. To address these limitations, recent advancements in three-dimensional (3D) NHP reproduction methods have been introduced [14,18]. While these methods offer improvements, they often require additional scanning processes or equipment, which may not eliminate all practical challenges. Third, the use of external devices or markers, such as customized bite jigs, orientation sensors, radiopaque stickers, or pose estimation algorithms like a pose from orthography and scaling with iterations (POSIT), can be uncomfortable and may alter natural facial expressions [19,20,21]. These devices require additional installation procedures and are cumbersome and complex. This can lead to errors in surgical planning and compromise patient comfort [22]. Finally, methods involving additional equipment or procedures, like 3D scanners or gyroscope sensors, increase time consumption, costs, and patient burden [23]. Extra scanning processes with additional surgical planning times require significant financial investment, which may not be feasible in all clinical settings. Therefore, an automatic and accurate method is required to reproduce NHP on CT scans from orthognathic patients.

Recent advances in deep-learning-based head pose estimation have significantly impacted fields such as computer vision and human–machine interaction, demonstrating precise head pose estimation techniques from images and videos for applications like gaze tracking, facial recognition, and virtual reality [24,25,26]. These advancements have shown that deep learning models can accurately predict head poses under various conditions, suggesting their potential applicability to medical fields [24,27,28]. As far as we know, however, no previous studies have reproduced NHP in CT scans to improve the accuracy of orthognathic surgery using deep learning.

The purpose of this study is to improve the accuracy of orthognathic surgery by automatically reproducing NHP in CT scans using a geometric deep learning network (NHP-Net). We developed NHP-Net that took a point cloud input from the skull mesh and regressed 3 × 3 rotation matrix elements to reproduce NHP directly. Our contributions include the following: (1) we developed a geometric deep learning network named NHP-Net that predicts rotation matrix elements of NHP in the CT scan of an orthognathic patient; (2) we conducted comprehensive experiments to assess the effectiveness of the size of points and loss functions.

## 2. Materials and Methods

### 2.1. Data Acquisition and Preparation

We included 150 patients (87 females and 63 males; mean age 28.42 ± 8.53 years) who underwent orthognathic surgeries at Seoul National University Dental Hospital from 2018 to 2023. The cohort was selected to represent a broad range of clinical presentations typical of orthognathic surgery cases. Specifically, patients with Class II and Class III malocclusions as well as various mandibular shapes were included to ensure diversity in anatomical features. However, patients with severe craniofacial anomalies beyond common skeletal discrepancies or those who had previously undergone extensive facial surgeries were excluded to reduce variability and maintain model robustness. This selection strategy aimed to create a dataset representative of most orthognathic surgery cases, ensuring that the model’s performance would be applicable to typical clinical scenarios. CT scans from patients were obtained using a multi-detector computed tomography (SOMATOM Sensation 10, Siemens, Munich, Germany) operating at 120 kVp and 80 mA, with a slice thickness of 0.75 mm. All patient data were anonymized to protect privacy. The NHP for each patient was manually annotated by an oral and maxillofacial surgeon using commercial software (Mimics Research 19.0, Materialise, Leuven, Belgium). Maxillofacial bone areas were segmented using the global thresholding technique in CT scans, and the segmented maxillofacial bone areas were converted into skull mesh data (STL format). Specifically, global thresholding involves segmenting bone regions based on CT intensity values measured in Hounsfield units (HU). Let *I*(*x*, *y*, *z*) represent the CT intensity at voxel (*x*, *y*, *z*), and *T* (in HU) be the empirically determined threshold value. The segmented volume *S* is defined as

(1) $$S(x,y,z)=\left\{\begin{array}{c} 1,\;\;if\;I\left(x,\;y,\;z\right)\geq T\\ 0,\;\;if\;I\left(x,\;y,\;z\right)<T \end{array}\right.$$

Only voxels with intensities above *T* are considered part of the bone. This thresholding ensures accurate extraction of bone structures, which are then converted into 3D skull meshes for subsequent processing by NHP-Net. We uniformly sampled 2048 point clouds from each skull mesh data, and each point cloud input was normalized by shifting the centroid of the center points to the origin [29,30]. We chose to represent the skull geometry as a point cloud rather than using the raw mesh format. This approach eliminates the need to handle irregular mesh connectivity and ensures a uniform input structure. By sampling a fixed number of points, we maintain a consistent data representation, allowing NHP-Net to focus on orientation prediction without being influenced by varying mesh quality or resolution. The CT dataset was divided into training, validation, and test sets, consisting of 90, 30, and 30 patients, respectively.

### 2.2. Network Architecture of NHP-Net

**Figure 2:**
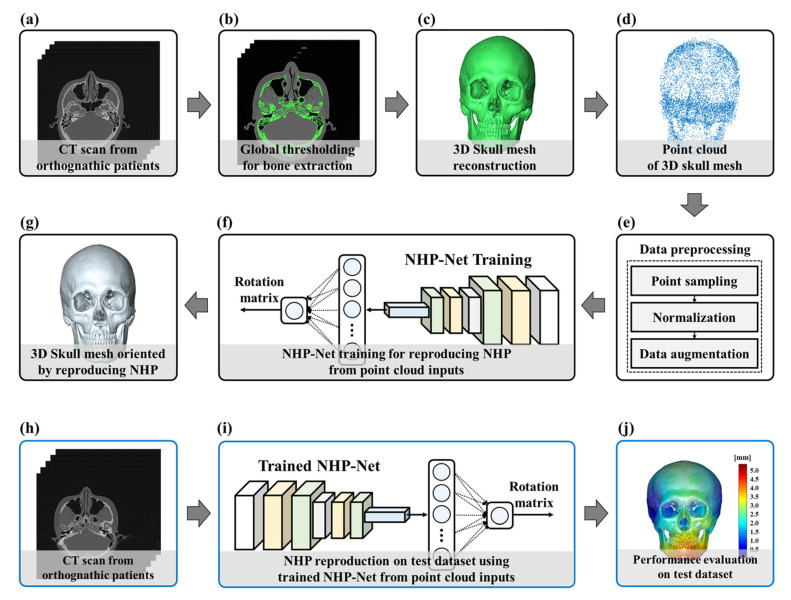
The schematic diagram of our proposed method. (**a**) CT scans from orthognathic patients on the training dataset. (**b**) Global thresholding for maxillofacial bone extraction. (**c**) Three-dimensional skull mesh reconstruction of maxillofacial bone area. (**d**) Point cloud extraction from 3D skull mesh. (**e**) Data preprocessing for deep learning. (**f**) NHP-Net training. (**g**) Three-dimensional skull mesh oriented by reproducing NHP from NHP-Net. (**h**) CT scans from orthognathic patients on the test dataset. (**i**) NHP reproduction on the test dataset using trained NHP-Net. (**j**) Evaluation process of the NHP-Net.

**Figure 3:**
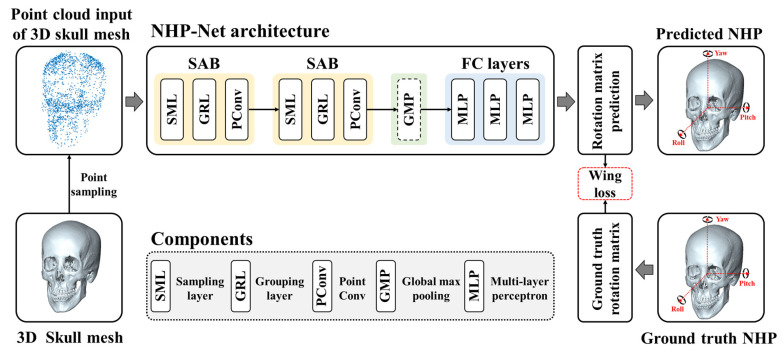
Illustration of our NHP-Net architecture. NHP-Net takes a point cloud input of the 3D skull mesh and regresses a 3 × 3 rotation matrix for reproducing NHP.

In this study, we proposed a geometric deep learning network (NHP-Net) to automatically reproduce NHP from CT scans, as shown in Figure 2. NHP-Net took a point cloud input from the skull mesh and regressed 3 × 3 rotation matrix elements to reproduce NHP. Our NHP-Net has two set abstraction blocks (SAB), a global max-pooling (GMP), and fully connected (FC) layers, as shown in Figure 3. Each SAB progressively downsamples the point cloud while extracting local and global geometric features at multiple scales. To achieve this, each SAB performs three key operations: sampling, grouping, and convolution [29,30,31]. Sampling employs iterative farthest point sampling (FPS) to select centroids from the point cloud, ensuring uniform coverage of the 3D space. For a given input point set *P*, FPS selects a subset *P*′ of centroids that are maximally distant from each other. This is achieved using the following selection criterion at iteration *k*:

(2) $${p^{\prime }}_{k}=\underset{p\;\in P}{\mathrm{argmax}}d(p,P\prime )$$

Grouping then identifies points within a radius *r* around each centroid *p*′_*i*_, forming local point groups *G*_*i*_ as:

(3) $$G_{i}=\{\;p_{j}\in P|\;\left\|p_{j}\right.-\left.{p^{\prime }}_{i}\right\|\leq r\;\}$$

These groups define the local regions from which geometric features are extracted.

Convolution is achieved using the PointConv operation, which generalizes convolution to point clouds. For each centroid *p*′_*i*_ and its local neighborhood *G*_*i*_, the updated feature *f*′*_i_* is calculated as:

(4) $${f\prime }_{i}=\sum_{p_{j}\in G_{i}}\;\frac{{W\left(p_{j}-{p^{\prime }}_{i}\right)\cdot f}_{j}}{\rho (p_{j})}$$

Here, *W*(*p*_*j*_ − *p*′_*i*_) is a learnable convolutional kernel defined by a multi-layer perceptron (MLP), and *ρ*(*p*_*j*_) adjusts for varying point densities. This approach captures local geometric relations and corrects for non-uniform distributions of points.

By employing these operations twice within the two SABs, NHP-Net progressively downsamples the point cloud, extracting hierarchical features at multiple levels. The final features are aggregated using a global max-pooling layer, and the fully connected (FC) layers predict the 3 × 3 rotation matrix for NHP reproduction. This hierarchical feature learning enables NHP-Net to capture both local and global geometric features, resulting in superior rotation matrix prediction accuracy. Overall, the model comprises approximately 1.46 million learnable parameters.

We employed the Wing loss (WL) function to handle both small and large errors effectively in network training. WL is particularly well-suited for angle regression tasks because it combines the advantages of both L1 and L2 loss functions, providing robustness to outliers while maintaining sensitivity to minor errors [32]. This characteristic is crucial for accurately predicting roll, pitch, and yaw angles in natural head position estimation. WL is defined as

(5) $$WL\left(x,y\right)=\left\{\begin{array}{c} \omega ln\left(1+\left|x-y\right|/\varepsilon \right)\;\;\;\;\;\;\;\;\;\;\;if\;\left|x-y\right|<\omega \\ \left|x-y\right|-C\;\;\;\;\;\;\;\;\;\;\;\;\;\;\;\;\;\;\;\mathrm{ortherwise} \end{array}\right.$$

where x and y are predicted 3 × 3 rotation matrix and ground truth of the 3 × 3 rotation matrix, respectively. ω, ε, and C are hyperparameters in WL, which control the smoothness of the loss curve, where C is defined as ω−ωln⁡(1+ω∕ε). In this work, we set ω=10 and ε=3  in WL, achieving the best performance.

In the inference stage, the trained NHP-Net is used to estimate the reproducing NHP of CT scans from an orthognathic patient. We uniformly sampled point clouds on the input skull mesh and normalized them. Then, NHP-Net predicted the 3×3 rotation matrix for reproducing NHP. To ensure the orthogonality of the predicted 3×3 rotation matrix, we applied singular value decomposition (SVD) [33,34]. The output matrix R′ was decomposed into $U\Sigma V^{⊤}$, and the refined rotation matrix R was obtained as $R=UV^{⊤}$. Subsequently, the refined rotation matrix R was applied to the vertices of the skull mesh to align it to the NHP.

**Figure 4:**
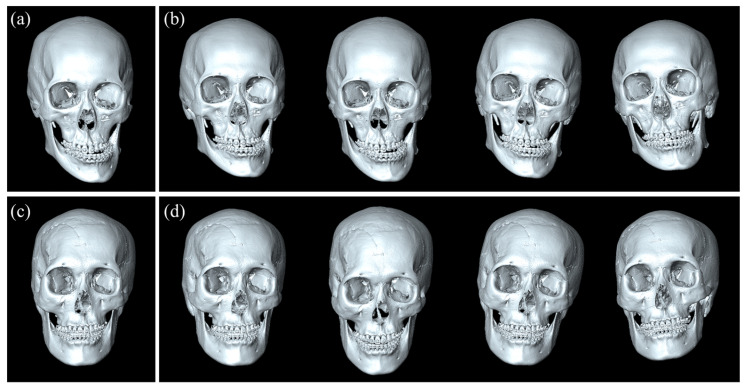
Illustrations of the data augmentation samples. The original 3D skull meshes (**a**,**c**) are augmented by randomly varying the roll, pitch, and yaw angles within ±50% of their original values, resulting in multiple augmented samples (**b**,**d**) used for training NHP-Net.

We used the Adam optimizer with an initial learning rate of 0.0001, where the learning rate was decreased to half of it every 50 epochs. The model was trained for 500 epochs with a batch size of 1 using a single NVIDIA Titan RTX GPU with 24 GB (NVIDIA Corporation, Santa Clara, CA, USA). We implemented NHP-Net architecture using Python3 and PyTorch 1.9.0. To enhance the robustness and generalizability of NHP-Net, we applied data augmentation techniques to the training dataset. Specifically, we increased the number of the training set by 30 times. Each augmented sample was created by randomly varying the original NHP roll, pitch, and yaw angles within ±50% of their original values. We chose this variation range to introduce significant diversity while ensuring the altered angles remained within physiologically plausible limits. By maintaining the average roll, pitch, and yaw angles of the data augmented dataset equal to those of the original data, we preserved the overall statistical properties, preventing any bias in the model training. This approach generated a total of 2700 training samples from the original 90 samples after data augmentation. Figure 4 illustrates augmented samples.

### 2.3. Evaluation Metrics

To evaluate the performance of deep learning models, we employed mean absolute error (MAE) and rotation error (RE), which were commonly used in regression tasks involving angular measurements. MAE assesses the average magnitude of the errors in predicting the individual roll, pitch, and yaw angles compared to the ground truth. For each angle, MAE is calculated as

(6) $${\mathrm{MAE}}_{\mathrm{angle}}=\frac{1}{n}\sum_{i=1}^{n}\left|{\hat{\theta }}_{i}-\theta_{i}\right|$$

where ${\hat{\theta }}_{i}$ is the is the predicted value of the angle (roll, pitch, or yaw) for the i-th sample, $\theta_{i}$ is the ground truth value of the same angle for the i-th sample, and n is the number of samples. The MAE is calculated separately for each angle. RE measures the angular difference between the predicted rotation matrix $\hat{R}\;$and the ground truth rotation matrix R. It is calculated using the following formula:

(7) $$RE={cos}^{-1}\left(\frac{Tr\left(\hat{R}R^{⊤}\right)-1}{2}\right)$$

where Tr(·) denotes the trace of a matrix. To determine the statistical significance of our results, we performed paired *t*-tests comparing the MAE for each angle value of NHP-Net with those of other deep learning models, specifically PointNet, DGCNN, and Point Cloud Transformer. A *p*-value less than 0.05 was considered statistically significant. All statistical analyses were conducted using SPSS Statistics ver. 21 (SPSS Inc., Chicago, IL, USA).

## 3. Results

Table 1 shows the performance of NHP-Net using WL (*ω* = 10 and *ε* = 3) with varying numbers of point cloud points (512, 1024, 2048, and 4096). As the number of points increased, the MAEs for roll, pitch, yaw, and RE generally decreased. Notably, at 2048 points, the RE was the lowest at 1.918° ± 1.099°, with MAE for roll at 0.665° ± 0.530° and MAE for yaw at 0.810° ± 0.786°, being the lowest among all configurations. Although the MAE for pitch at 2048 points (1.246° ± 1.155°) was slightly higher than at 1024 points (1.142° ± 0.983°), the overall performance at 2048 points was optimal due to the lowest RE and MAEs for roll and yaw. However, at 4096 points, the RE slightly increased to 2.026° ± 0.943°, indicating a slight performance degradation. This suggests that increasing the number of points beyond 2048 does not lead to significant performance improvement and may even cause degradation in some angles. Therefore, 2048 points are considered the optimal number in terms of efficiency and overall performance.

Table 2 presents the results of a comparison of various loss functions (WL, MAE loss, MSE loss, and Huber loss) in NHP-Net while fixing the number of point cloud points at 2048. When using WL (*ω* = 10 and *ε* = 3), MAEs for roll, pitch, and yaw were 0.665° ± 0.530°, 1.246° ± 1.155°, and 0.810° ± 0.786°, respectively, with the lowest RE of 1.918° ± 1.099°. Notably, the absolute error for pitch was lowest when using MAE loss (1.226° ± 0.962°), and the absolute error for yaw was lowest when using MSE loss (0.749° ± 0.632°). The REs for MAE, MSE, and Huber losses were 2.033° ± 1.007°, 1.970° ± 0.922°, and 2.056° ± 1.023°, respectively. Although WL showed slightly better performance, the overall differences in performance across loss functions were insignificant.

Table 3 displays the performance of NHP-Net according to various combinations of hyperparameters *ω* and *ε* in WL. The experimental results indicated that with the combination of *ω* = 10 and *ε* = 3.0 in WL, the MAEs for roll, pitch, and yaw were 0.665° ± 0.530°, 1.246° ± 1.155°, and 0.810° ± 0.786°, respectively, and the RE was the lowest at 1.918° ± 1.099°. Overall, this combination provided the lowest RE and superior performance across all angles, leading us to select *ω* = 10 and *ε* = 3.0 as the optimal hyperparameters for the WL.

**Figure 5:**
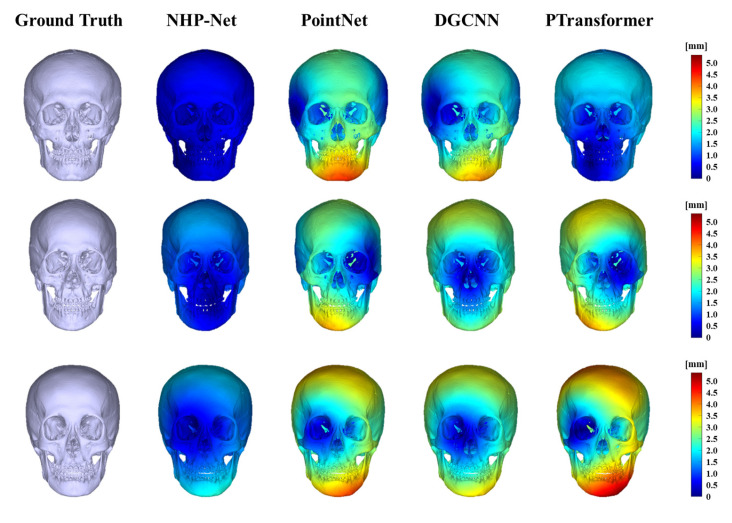
Surface distance errors between 3D skull meshes oriented by ground truth natural head position (NHP) and NHP reproduced by different deep learning models in the frontal view. Distributions of surface distance errors are visualized with a color map, where blue indicates low surface distance error and red indicates high surface distance. NHP-Net demonstrates the most accurate alignment with the reference, as shown by the lower surface distance errors than other deep learning models.

**Figure 6:**
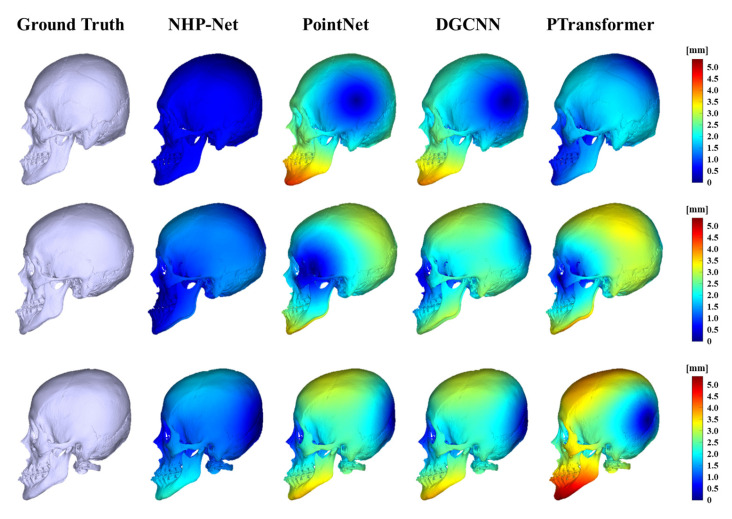
Surface distance errors between 3D skull meshes oriented by ground truth natural head position (NHP) and NHP reproduced by different deep learning models in the side view. Distributions of surface distance errors are visualized with a color map, where blue indicates low surface distance error and red indicates high surface distance. NHP-Net demonstrates the most accurate alignment with the reference, as shown by the lower surface distance errors than other deep learning models.

**Figure 7:**
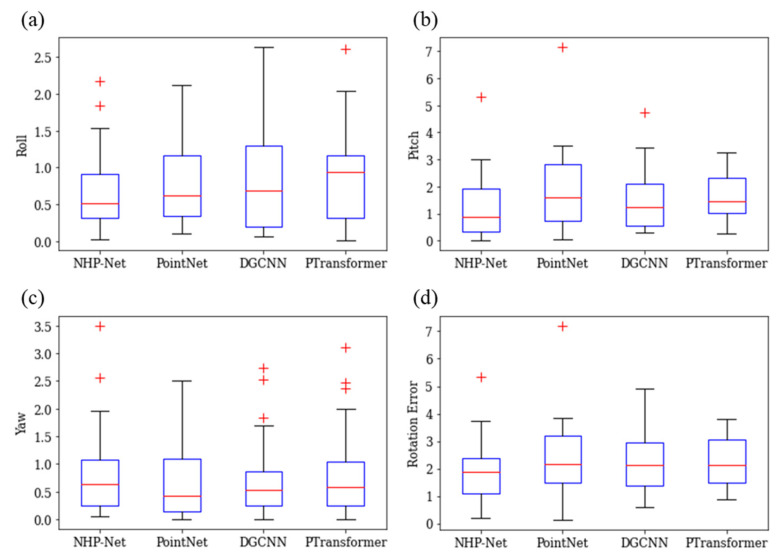
Boxplots of mean absolute error performance results for (**a**) roll, (**b**) pitch, (**c**) yaw, and (**d**) rotation error from different deep learning models. Each box represents the first and third quartiles of the data, with the median indicated by a red line inside the box. Whiskers extend above and below each box to ±1.5 times the interquartile range (IQR), and outliers beyond 1.5 IQR are visualized as red ‘+’ marks.

Table 4 shows the quantitative performance comparison results from different deep learning models. The number of point cloud points was fixed at 2048, and the loss function was set to WL (*ω* = 10 and *ε* = 3.0). NHP-Net achieved MAEs of 0.665° ± 0.530° for roll, 1.246° ± 1.155° for pitch, and 0.810° ± 0.786° for yaw, with the lowest RE of 1.918° ± 1.099°. The REs for other models (PointNet, DGCNN, and PTransformer) were 2.432° ± 1.305°, 2.295° ± 1.036°, and 2.238° ± 0.855°, respectively. Statistical significance tests showed that NHP-Net had significantly lower MAEs in roll, pitch, and RE compared to the other deep learning models (*p* < 0.05). However, for yaw, the difference was not statistically significant (*p* > 0.05). Additionally, comparisons of the surface distance errors in Figure 5 and Figure 6 demonstrated that NHP-Net produced results most similar to the actual angles, suggesting that the model has high prediction accuracy. In Figure 7, we visually confirmed that NHP-Net had a lower median error and a narrower distribution range, indicating stable and consistent performance.

To evaluate potential model bias regarding gender, we performed a subgroup analysis. For male patients (*n* = 9), the average MAEs were 0.694° ± 0.422° (roll), 1.093° ± 0.788° (pitch), and 0.906° ± 0.854° (yaw), with an RE of 2.083° ± 1.090°. For female patients (*n* = 21), the average MAEs were 0.653° ± 0.570° (roll), 1.312° ± 1.275° (pitch), and 0.769° ± 0.751° (yaw), with an RE of 1.847° ± 1.095°. Independent *t*-tests showed no significant differences across genders (*p* > 0.05 for all metrics), indicating that the model’s performance is consistent regardless of patient gender.

**Figure 8:**
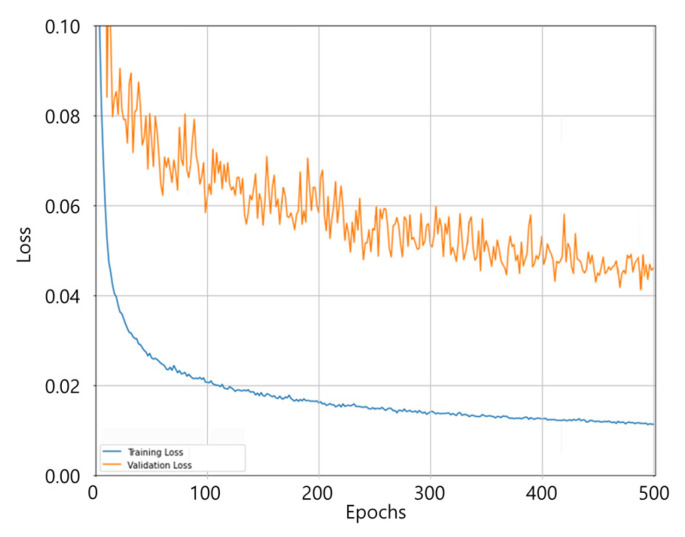
Wing loss during the training of the NHP-Net model with a batch size of 1. The training loss decreased from 0.15925 to 0.01563, and the validation loss decreased from 0.31455 to 0.04625 over the training epochs.

Figure 8 illustrates the progression of the Wing loss during training and validation, demonstrating a significant reduction in both losses over the epochs. The training loss decreased from 0.15925 to 0.01563, and the validation loss decreased from 0.31455 to 0.04625, indicating stable convergence and effective learning of the NHP-Net model.

## 4. Discussion

Traditional techniques often suffer from reproducibility issues due to individual variations and the difficulty for patients to maintain a consistent natural posture [9]. Additionally, methods requiring external devices or markers can be uncomfortable and may alter natural facial expressions, leading to potential inaccuracies in surgical planning [19,20,21]. With the advent of deep learning and its success in head pose estimation in other fields, we introduced a deep learning approach utilizing geometric representations from point clouds of skull meshes to predict a rotation matrix of NHP from a CT scan. In this study, we aimed to develop a geometric deep learning network for reproducing NHP in CT scans from orthognathic patients. To the best of our knowledge, this is the first study to utilize deep learning techniques for reproducing NHP directly from CT scans. Our results demonstrate that NHP-Net outperformed other state-of-the-art deep learning models, such as PointNet, DGCNN, and Point Cloud Transformer, in terms of RE and MAEs for roll and pitch angles. Specifically, NHP-Net achieved the lowest RE of 1.918° ± 1.099°; significantly lower MAEs in roll and pitch; and a significantly lower RE compared to the other models (*p* < 0.05). The superior performance of NHP-Net over PointNet, DGCNN, and Point Cloud Transformer stems from key improvements in design and training. First, NHP-Net employs two set abstraction blocks (SABs) to downsample the point cloud while capturing hierarchical features. Unlike DGCNN’s edge connections and PointNet’s independent point processing, SABs enable NHP-Net to model both local and global geometric features crucial for NHP prediction. Compared to the self-attention of Point Cloud Transformer, SABs explicitly capture local geometric relationships, better representing the skull’s detailed curvature. Second, the WL function enhances robustness by balancing sensitivity to small errors and resistance to large errors, outperforming MSE and MAE loss. This approach improves precision and robustness in NHP estimation, particularly in roll and pitch angles, which are critical for surgical planning. We employed two SAB blocks as this configuration balanced complexity and performance. Preliminary experiments indicated that increasing the number of SAB blocks did not yield meaningful improvements in accuracy, while potentially increasing computational cost and the risk of overfitting (Table 5).

Despite experimenting with various loss functions, including MAE loss, MSE loss, Huber loss, and WL, our results showed that the overall performance differences among them were insignificant (Table 2). This minimal variance could be attributed to the robustness of the network architecture, which effectively captures the features necessary for NHP estimation regardless of the specific loss function used. The similarity in performance suggests that the model’s capacity to learn the underlying data distribution and the rotational relationships is strong enough that the choice of loss function has a marginal impact on the final outcome. We chose WL as the loss function for several reasons. First, WL demonstrated the lowest RE among all tested loss functions, indicating a slight advantage in overall performance. Second, WL is specifically designed to handle both small and large errors effectively by combining the benefits of L1 and L2 losses [32]. This property is particularly beneficial in regression tasks involving angles, where outliers or extreme values can adversely affect model training. By using WL, the model becomes more robust to such outliers, potentially improving its generalization to unseen data. Moreover, the asymptotic nature of WL near zero error makes the model more sensitive to small deviations, which is crucial for precise NHP reproduction. In medical applications, even minor errors can have significant clinical implications. Therefore, opting for a loss function that prioritizes accuracy in small error regions aligns with the goal of achieving high precision in surgical planning. By employing 2048 point clouds and optimizing the hyperparameters of the WL function (*ω* = 10 and *ε* = 3.0), we balanced computational efficiency with model accuracy. The superior performance of NHP-Net can also be attributed to its ability to effectively capture local and global features of point clouds of 3D skull meshes using geometric deep learning.

Compared to traditional methods for NHP reproduction, which often require additional equipment or manual intervention [21,23], our approach offers a fully automatic solution that operates solely on CT data. This reduces the time and cost associated with NHP determination and minimizes patient discomfort and potential errors arising from manual procedures. Unlike conventional methods requiring manual adjustments, fiducial markers, or specialized devices, NHP-Net operates solely on standard CT data, eliminating the need for external equipment. By automating NHP determination, NHP-Net reduces preoperative planning time and clinician intervention. For instance, conventional methods requiring fiducial markers or bite jigs can add 10–20 min per patient, where-as NHP-Net performs the process automatically after CT acquisition. This not only im-proves patient comfort but also minimizes procedural costs and operational complexity. Additionally, NHP-Net can be seamlessly integrated into existing hospital workflows, utilizing standard workstations with GPUs, thereby avoiding the need for substantial infra-structure changes. Our findings align with recent advancements in deep-learning-based head pose estimation in computer vision [24,25,26], extending their applicability to the medical field. We expect that NHP-Net will help to improve the workflow in orthognathic surgery.

Our study had several limitations. First, the CT dataset used for deep learning was relatively small, which may limit the generalizability of the model. To address this, future research will focus on assembling larger, more diverse datasets from multiple institutions, including hospitals from different regions and patient populations, to improve the model’s robustness and applicability. Second, the ground truth NHP was determined by a single oral and maxillofacial surgeon. To reduce observer bias, future studies will incorporate annotations from multiple surgeons using consensus-based methods, such as inter-observer agreement analysis and majority voting. Beyond orthognathic surgery, NHP-Net has potential applications in other craniofacial procedures, such as maxillofacial trauma and cranial surgeries, where accurate orientation is crucial. Prospective clinical trials with heterogeneous datasets will further validate and refine the model, ultimately facilitating its adoption in diverse clinical environments. Third, our approach involves converting the 3D skull mesh into a point cloud. This conversion standardizes the input format and ensures consistent data dimensions but may result in the loss of some geometric details inherent in the raw mesh [35]. Future work could explore hybrid approaches that retain mesh connectivity information while leveraging the advantages of point cloud representations. Last, while NHP-Net showed significantly lower errors in roll, pitch, and RE compared to the other models, the improvement in yaw was not statistically significant (*p* > 0.05). NHP-Net may require further fine-tunings to enhance reproduction accuracy in yaw angles.

## 5. Conclusions

In this study, we introduced NHP-Net, a geometric deep learning network designed to automatically reproduce NHP in CT scans from orthognathic patients. By leveraging SAB, GMP, and FC layers and optimizing hyperparameters of the WL function, NHP-Net effectively captured both local and global geometric features of point clouds of 3D skull meshes. Our experimental results demonstrate that NHP-Net outperformed other state-of-the-art deep learning models, achieving the lowest RE of 1.918° ± 1.099° and significantly lower MAEs in roll and pitch angles (*p* < 0.05). We expected that our proposed NHP-Net could be a significant advancement in automatic NHP reproduction from CT scans to reduce the workload of surgeons for orthognathic surgery planning and improve surgical precision and patient outcomes.

## Figures and Tables

**Table 1:** Quantitative performance from NHP-Net with different point cloud sizes. Mean absolute error is used to evaluate performance for roll, pitch, and yaw.

The Number of Points	Roll (°)	Pitch (°)	Yaw (°)	Rotation Error (°)
512	1.178 ± 0.905	1.306 ± 1.343	1.861 ± 1.322	3.007 ± 1.372
1024	0.959 ± 0.821	0.917 ± 0.936	1.212 ± 1.144	2.150 ± 1.212
2048	0.665 ± 0.530	1.246 ± 1.155	0.810 ± 0.786	1.918 ± 1.099
4096	0.764 ± 0.572	1.310 ± 0.897	0.940 ± 0.823	2.026 ± 0.943

Values are presented as mean ± standard deviation.

**Table 2:** Quantitative performance from NHP-Net with different loss functions. Mean absolute error is used to evaluate performance for roll, pitch, and yaw.

	Roll (°)	Pitch (°)	Yaw (°)	Rotation Error (°)
WL (*ω* = 10 and *ε* = 3)	0.665 ± 0.530	1.246 ± 1.155	0.810 ± 0.786	1.918 ± 1.099
MAE loss	0.865 ± 0.465	1.226 ± 0.962	0.894 ± 0.978	2.033 ± 1.007
MSE loss	0.796 ± 0.552	1.329 ± 1.033	0.749 ± 0.632	1.970 ± 0.922
Huber loss	0.823 ± 0.623	1.329 ± 1.100	0.786 ± 0.654	2.056 ± 1.023

Values are presented as mean ± standard deviation.

**Table 3:** Quantitative performance from NHP-Net with different *ω* and *ε* hyperparameters in Wing loss. Mean absolute error is used to evaluate performance for roll, pitch, and yaw.

*ω*	*ε*	Roll (°)	Pitch (°)	Yaw (°)	Rotation Error (°)
5	0.5	0.788 ± 0.651	1.333 ± 1.020	0.782 ± 0.784	2.024 ± 1.118
	1.0	0.925 ± 0.664	1.292 ± 1.087	0.755 ± 0.714	2.038 ± 1.129
	2.0	0.870 ± 0.724	1.342 ± 1.143	0.719 ± 0.745	2.044 ± 1.146
	3.0	0.820 ± 0.600	1.289 ± 1.107	0.750 ± 0.697	1.994 ± 1.051
	4.0	0.855 ± 0.597	1.515 ± 1.015	0.882 ± 0.773	2.254 ± 1.077
10	0.5	0.817 ± 0.579	1.229 ± 0.992	0.836 ± 0.756	1.996 ± 1.047
	1.0	1.051 ± 0.804	1.323 ± 0.906	1.024 ± 1.014	2.328 ± 1.123
	2.0	0.858 ± 0.624	1.201 ± 0.878	0.820 ± 0.654	1.928 ± 0.851
	3.0	0.665 ± 0.530	1.246 ± 1.155	0.810 ± 0.786	1.918 ± 1.099
	4.0	0.808 ± 0.591	1.272 ± 1.040	0.802 ± 0.685	2.014 ± 1.032

Values are presented as mean ± standard deviation.

**Table 4:** Quantitative performance from different deep learning models. Mean absolute error is used to evaluate performance for roll, pitch, and yaw.

	Roll (°)	Pitch (°)	Yaw (°)	Rotation Error (°)
NHP-Net	0.665 ± 0.530	1.246 ± 1.155	0.810 ± 0.786	1.918 ± 1.099
PointNet	0.825 ± 0.583 *	1.875 ± 1.480 *	0.715 ± 0.677	2.432 ± 1.305 *
DGCNN	0.934 ± 0.689 ^†^	1.565 ± 1.081 ^†^	0.805 ± 0.679	2.295 ± 1.036 ^†^
PTransformer	0.854 ± 0.613 ^‡^	1.614 ± 0.793 ^‡^	0.867 ± 0.792	2.238 ± 0.855 ^‡^

Values are presented as mean ± standard deviation. * Significant difference between NHP-Net and PointNet (*p* < 0.05). ^†^ Significant difference between NHP-Net and DGCNN (*p* < 0.05). ^‡^ Significant difference between NHP-Net and PTransformer (*p* < 0.05).

**Table 5:** Performance and training time comparison using different numbers of SAB blocks. Performance is evaluated using mean absolute error (MAE) for roll, pitch, yaw, and rotation error. Training time is reported per epoch.

Number of SAB	Roll (°)	Pitch (°)	Yaw (°)	Rotation Error (°)	Training TIME (min/epoch)
1	0.712 ± 0.610	1.342 ± 1.200	0.845 ± 0.790	2.145 ± 1.130	5.2
2	0.665 ± 0.530	1.246 ± 1.155	0.810 ± 0.786	1.918 ± 1.099	6.0
3	0.672 ± 0.520	1.230 ± 1.140	0.806 ± 0.780	1.913 ± 1.095	8.5

Values are presented as mean ± standard deviation.

## Data Availability

The datasets generated and/or analyzed during the current study are not publicly available due to the restriction by the Institutional Review Board (IRB) of Seoul National University Dental Hospital in order to protect patient’s privacy but are available from the corresponding author on reasonable request.
